# Genome editing reveals *dmrt1* as an essential male sex-determining gene in Chinese tongue sole (*Cynoglossus semilaevis*)

**DOI:** 10.1038/srep42213

**Published:** 2017-02-16

**Authors:** Zhongkai Cui, Yun Liu, Wenwen Wang, Qian Wang, Ning Zhang, Fan Lin, Na Wang, Changwei Shao, Zhongdian Dong, Yangzhen Li, Yingming Yang, Mengzhu Hu, Hailong Li, Fengtao Gao, Zhanfei Wei, Liang Meng, Yang Liu, Min Wei, Ying Zhu, Hua Guo, Christopher H. K. Cheng, Manfred Schartl, Songlin Chen

**Affiliations:** 1 Key Lab of Sustainable Development of Marine Fisheries, Ministry of Agriculture; Yellow Sea Fisheries Research Institute, Chinese Academy of Fishery Sciences, Qingdao 266071, China; 2 Laboratory for Marine Fisheries Science and Food Production Processes, Qingdao National Laboratory for Marine Science and Technology, Qingdao 266273, China; 3 College of Fisheries and Life, Shanghai Ocean University, Shanghai 201306, China; 4 School of Biomedical Sciences, The Chinese University of Hong Kong, Shatin, N.T., Hong Kong, China; 5 School of Biomedical Sciences Core Laboratory, The Chinese University of Hong Kong Shenzhen Research Institute, Shenzhen 518057, China; 6 Physiologische Chemie, University of Würzburg, Biozentrum, and Comprehensive Cancer Center Mainfranken, University Clinic Würzburg, D-97074 Würzburg, Germany; 7 Texas A&M Institute for Advanced Studies and Department of Biology, Texas A&M University, College Station, Texas 77843, USA

## Abstract

Chinese tongue sole is a marine fish with ZW sex determination. Genome sequencing suggested that the Z-linked *dmrt1* is a putative male determination gene, but direct genetic evidence is still lacking. Here we show that TALEN of *dmrt1* efficiently induced mutations of this gene. The ZZ *dmrt1* mutant fish developed ovary-like testis, and the spermatogenesis was disrupted. The female-related genes *foxl2* and *cyp19a1a* were significantly increased in the gonad of the ZZ *dmrt1* mutant. Conversely, the male-related genes *Sox9a* and *Amh* were significantly decreased. The *dmrt1* deficient ZZ fish grew much faster than ZZ male control. Notably, we obtained an intersex ZW fish with a testis on one side and an ovary on the other side. This fish was chimeric for a *dmrt1* mutation in the ovary, and wild-type *dmrt1* in the testis. Our data provide the first functional evidence that *dmrt1* is a male determining gene in tongue sole.

Sex-determining (SD) genes are located on the sex chromosomes to initiate a series of signaling pathways of sex related events to induce the development of bipotential primordial gonads into testes or ovaries. So far, a number of sex-determining genes have been identified in several vertebrate species^1^. Besides the *SRY* gene in mammals^2^,^3^, a member of the Sox family of transcription factors, several other genes that were previously known to act in the regulatory network of sex determination and gonad development have been found to function as master sex determining gene, e.g. *dmrt1, Amh* or its receptor and others^4^,^5^,^6^,^7^,^8^.

Most of the identified sex determining genes are from species with a XY sex determination system, while much less is known about the sex determining genes in the ZW system. The Chinese half-smooth tongue sole (*C. semilaevis*) is a very important cultured marine flatfish with a ZW sex chromosome system^9^,^10^. With respect to identifying the master sex determining gene, tongue sole is an ideal model because the full genome was sequenced^11^, female specific AFLP and SSR molecular markers were discovered^12^,^13^, and high density genetic linkage groups were developed^14^,^15^. However, despite having a genetic sex determination by a well differentiated sex chromosome system, ~14% of ZW genetic females undergo sex-reversal to physiological males under normal rearing conditions, and relatively high temperature (28 °C) can increase the sex reversal rate of genetic females to ~73%^16^, which is similar to the phenomenon observed in Australian bearded dragon^17^.

Interestingly, our recent study suggested that the sex chromosome of tongue sole was derived from the same ancestral vertebrate protochromosome as the avian W and Z chromosomes^11^. Previous studies suggested that *dmrt1* located on the Z chromosome but absent from the W acts as a dosage-sensitive sex determining gene in chicken^4^. Also in *C. semilaevis dmrt1* is located on the Z chromosome and thus was considered to be an excellent candidate for the gene that determines male sexual development^11^. Only a corrupted version of *dmrt1* is present on the W and gene expression studies further supported its function as a master SD gene. However, functional evidence has not been provided so far.

Genome editing methods employing zinc-finger nucleases (ZFNs), transcription activator-like effector nucleases (TALENs) and clustered regulatory interspaced short palindromic repeats (CRISPR/Cas9) have been successively established and applied in freshwater fish such as zebrafish, medaka, rainbow trout and Nile tilapia^18^,^19^,^20^,^21^ making an important addition to the already well developed toolbox of producing genetically modified fish. On the contrary, a similar methodology has not been established for marine fish due to the lack of a feasible embryo microinjection method. For a single species, the Nibe croaker, stable introduction of a green fluorescence protein (GFP) transgene by embryo microinjection was achieved^22^. In particular genome editing techniques have not been successfully applied to marine fish.

In this study, we have for the first time developed an efficient protocol for microinjection of flatfish embryos and then constructed TALEN plasmids and developed genomic editing methods for tongue sole. With these techniques, we produced *dmrt1* mutants. Results provide functional evidence that *dmrt1* is the male sex-determining gene in Chinese tongue sole.

## Results

### Development of embryo microinjection technique in flatfish

To develop a microinjection technique in flatfish we first used a reporter plasmid (*psmyd1:gfp*) where GFP is expressed under control of a muscle specific promoter. Zygotes were microinjected within ~40 minutes post-fertilization beginning at 1-cell stage and up to 4-cell stage embryos.

**Figure 1:**
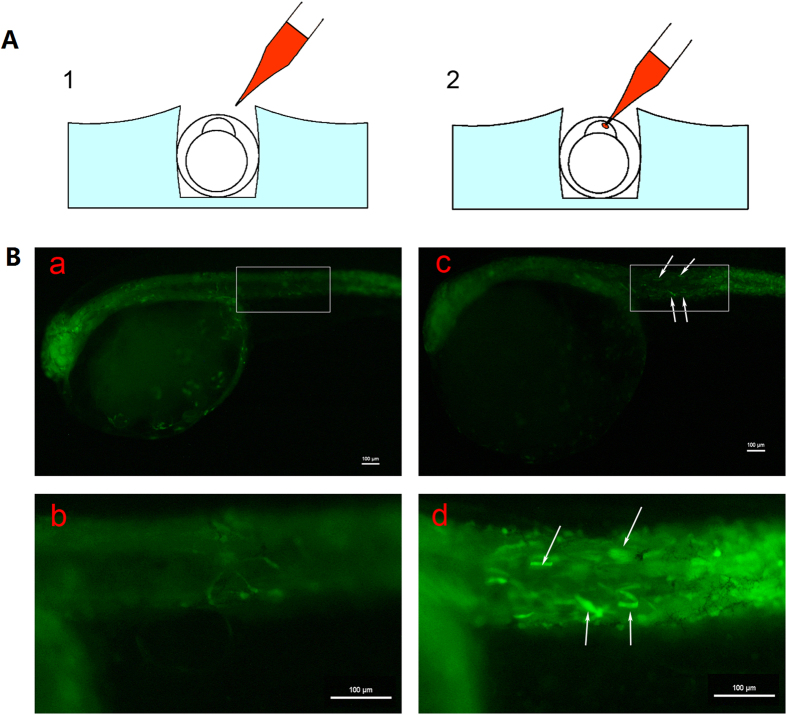
Microinjection method and transient expression of GFP in *C. semilaevis*. (**A**) Schematic drawing of microinjection. (**1)** A micropipette is passed through the chorion at an approximately 45-degree angle; (**2)** a droplet which is 1/10~1/5 of the cell volume is injected into the cell. (**B**) Transient expression of GFP in 2 dah *C. semilaevis*. (a) Control fish; (b) large magnification of frame area in (a); (c) transient expression of GFP in the muscles; (d) large magnification of frame area in (c) muscle fibres strongly expressing of GFP are indicated by arrows. Scale bar, 100 μm.

Successful injection was confirmed by expression of GFP in G0 fry. Of approximately 2000 injected embryos, nearly 500 embryos developed normally until gastrulation stage (~17 hpf). The survival rate of un-injected eggs is about 40%. At hatching stage, 46.2% (12/26) larvae showed clear GFP expression in skeletal muscle in accordance with the tissue-specificity of the *smyd1* promoter (Fig. 1B).

### TALENs effectively induce mutations in the *dmrt1* gene of *C. Semilaevis*

**Figure 2:**
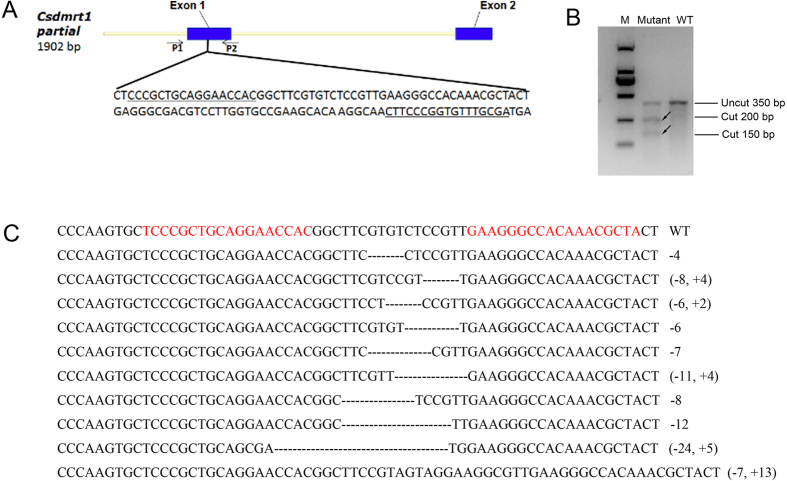
Genomic structure of the *C. semilaevis dmrt1* gene, design of *dmrt1*-TALENs and mutated sequences induced by TALENs. (**A**) *dmrt1*-TALENs designed to target exon 1 of the gene. (**B**) Detection of mutations in the injected embryos by *T7E* I digestion. (**C**) Mutant *dmrt1* sequences from TALEN injected embryos. The *T7E* I cut bands were recovered after gel electrophoresis and cloned for sequence analysis. “−” represents deletion of bp, “+” represents addition of bp. The numbers at the right side indicate the number of deleted or inserted base pairs.

To functionally test the role of *dmrt1* in sexual development of *C. semilaevis* a TALEN approach was used to eliminate *dmrt1* function. TALENs directed against exon1 sequences were injected. From 60,000 injected embryos, 500 survived until free swimming larvae stage and finally 65 adults were raised. In pooled microinjected embryos and larvae, mutagenic efficiency of *dmrt1* was about 55%. The *dmrt1* mutated samples in the *T7E* I tests were divided into two parts according to the 200 bp and 150 bp bands, respectively, recorded after *T7E* I digestion (Fig. 2B). From sequencing of embryos showing the target site to be mutated we recorded 10 different insertion and deletion events (Fig. 2C). Thus, the *dmrt1* TALENs used in this study are indeed suitable for targeted mutation of the *dmrt1* gene of *C. semilaevis* (Fig. 2).

The mutation frequency for survived TALEN sole was 50.77% (33/65). The 33 TALEN mutated sole consisted of 16 genetic females and 17 genetic males (Figure S2). There are a number of mutants with a mutation frequency ranging from 20% to 100% (Figure S3) and even different mutations were detected from the same individual. In general, the mutation rate of gonads was similar or even higher than that of somatic tissues (fins) (Figure S4).

### *Dmrt1-*deficient tongue sole have a sex-reversed phenotype and display female gene expression patterns

**Figure 3:**
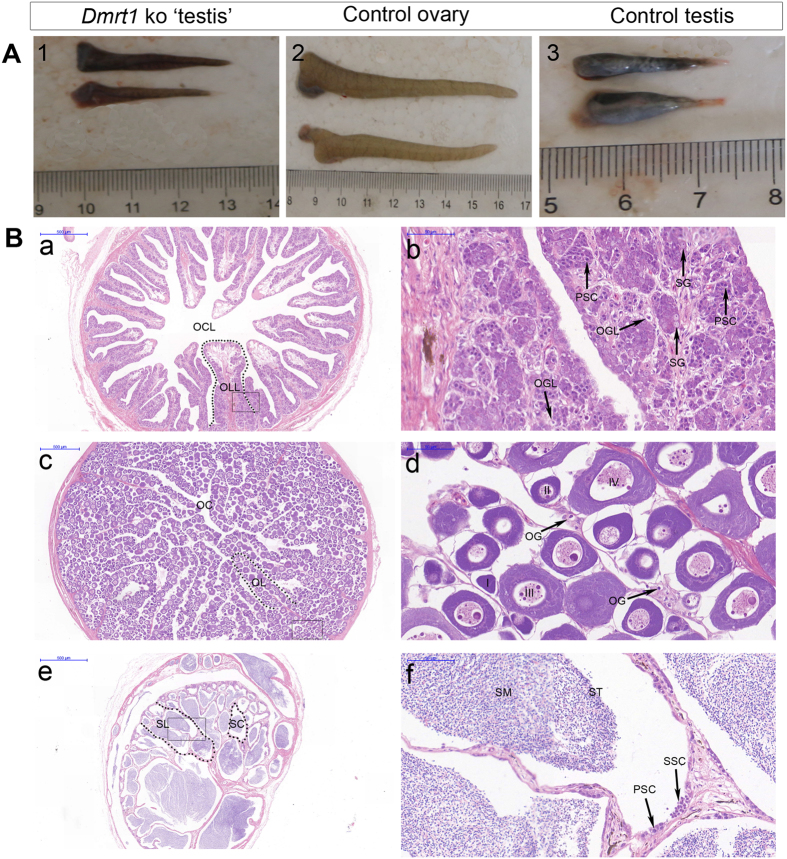
Effects of *dmrt1* disruption on gonad phenotype, sex differentiation. (**A**) Gross morphology of gonads from approximately one year old fish. (**1**) *dmrt1-*deficient ‘testes’; (**2**) wild-type ovaries; (**3**) wild-type testes. (**B**) histology of gonads from approximately one year old fish. (a) *dmrt1-*deficient testis. The development of testis is ceased. The shape of the *dmrt1-*deficient testes in transverse sections is similar to control ovaries, and there are structures resembling ovarian cavity and ovarian lamella in the gonad of the mutant male fish. ovarian cavity-like (OCL), ovarian lamella-like (OLL); (b) large magnification of frame area in a. No secondary spermatocytes, spermatids and sperm are observed. oogonia-like (OGL), spermatogonia (SG) and primary spermatocytes (PSC). (c) Ovary of control female, including ovarian cavity (OC), ovarian lamella (OL); (d) large magnification of frame area in (c). Four stages of oocytes: stage I - IV and oogonia (OG). (e) Testis of control male. seminiferous lobuli (SL), seminiferous cyst (SC); (f) larger magnification of frame area in (e). Secondary spermatocytes (SSC), spermatids (ST) and sperm (SM). Scale bar is shown in the figures.

In wild-type fish, testes are short and thick with a “cashew nut” shape, whereas ovaries are long, thin and have a circular shape. In *dmrt1-*deficient genotypic males, the gonads presented an ovary-like gross appearance (Fig. 3A) being significantly longer than the control testes. Histological examination of the gonads from one-year-old *dmrt1-*deficient fish revealed an abnormal structure. Compared to wild-type males, only a few spermatogonia, and few or in some cases even no spermatocytes and spermatids were observed (Fig. 3B,a and b). In addition, *dmrt1-*deficient testes showed structures typically found in normal ovaries, like ovarian lamella and a central lumen (Fig. 3B,a). Intriguingly, we detected in the mutant gonads structures that appeared to resemble oogonia (Fig. 3B,b).

**Figure 4:**
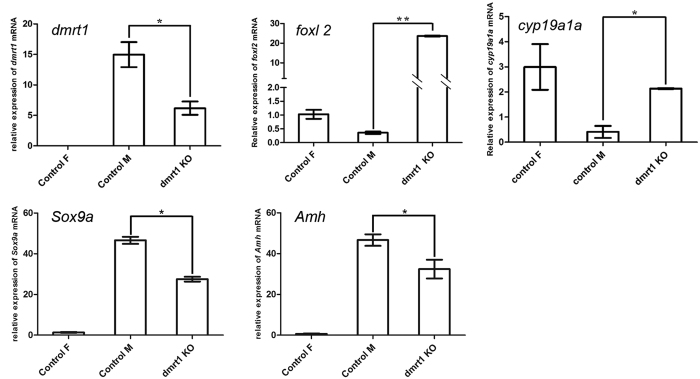
Gene expressions of sex differentiation markers on *dmrt1*-deficient gonad. *Dmrt1* and *foxl2* are key transcription factors in testicular and ovarian differentiation, respectively. *Cyp19a1a* encodes for aromatase which is responsible for estrogen production. *Sox9a* and *Amh* are male related genes. Relative mRNA expression of *dmrt1, foxl2, cyp19a1a, Sox9a* and *Amh* in *dmrt1*-deficient gonads at one year of age from one *dmrt1*-deficient and three wild-type gonads. *β-actin* was used for calibration.

*Dmrt1* gene expression analyses of mutant gonads revealed far less transcript abundance than samples from control males (Fig. 4 and Figure S5B). We then examined *foxl2* and *cyp19a1a* gene expression, which are expressed in ovary and essential for the maintenance of ovary differentiation^23^,^24^. And *Sox9a* and *Amh* gene expression were also examined. Compared to control males, the expression of *foxl2* and *cyp19a1a* was significantly increased in the *dmrt1*-deficient males (Fig. 4 and Figure S5A and C). Conversely, the male-related genes *Sox9a* and *Amh* were significantly decreased. Interestingly, the expression of *foxl2* in the *dmrt1*-deficient males was even significantly higher than in wild-type females (Fig. 4 and Figure S5C) (*p* < 0.05).

**Figure 5:**
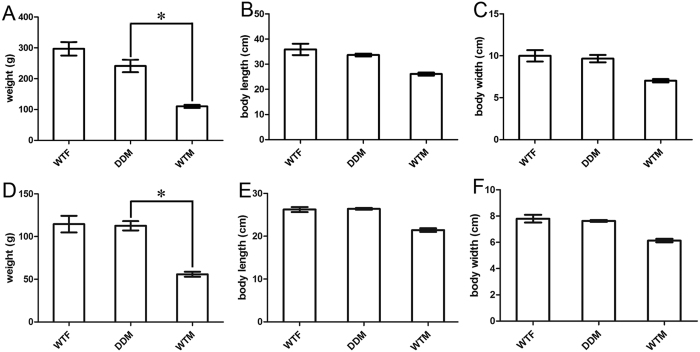
Growth parameters of *dmrt1*-deficient *C. semilaevis*. (**A**) Weight of *dmrt1-*deficient male *C. semilaevis* (DDM) in 2014 (1 year old) was significantly higher than of wild-type males (WTM) and similar to that of wild-type females (WTF). (**B**) Body length and (**C**) width of *dmrt1*-deficient *C. semilaevis* were larger than of wild-type male and close to the values of females. (**D**) Weight of *dmrt1-*deficient male *C. semilaevis* (DDM) in 2015 (8 months old) was also higher than of wild-type males (WTM) and similar to that of wild-type females (WTF). (**E**) Body length and (**F**) width of *dmrt1*-deficient *C. semilaevis* were larger than of wild-type male and close to the values of females.

The *dmrt1*-deficient males showed accelerated growth. Compared to wild-type males, the body weight of the *dmrt1*-deficient males was increased 2.5 fold (p < 0.01) (Fig. 5 and Table S2). The body width and length of the *dmrt1* deficient males were also larger than that of wild-type male fish (Fig. 5 and Table S2). These data indicate that disruption of *dmrt1* is connected to obvious changes in the growth characteristics of male *C. semilaevis*.

### Chimeric *dmrt1*-mutant fish develop as intersex with a wild-type testis and a *dmrt1* mutant ovary

**Figure 6:**
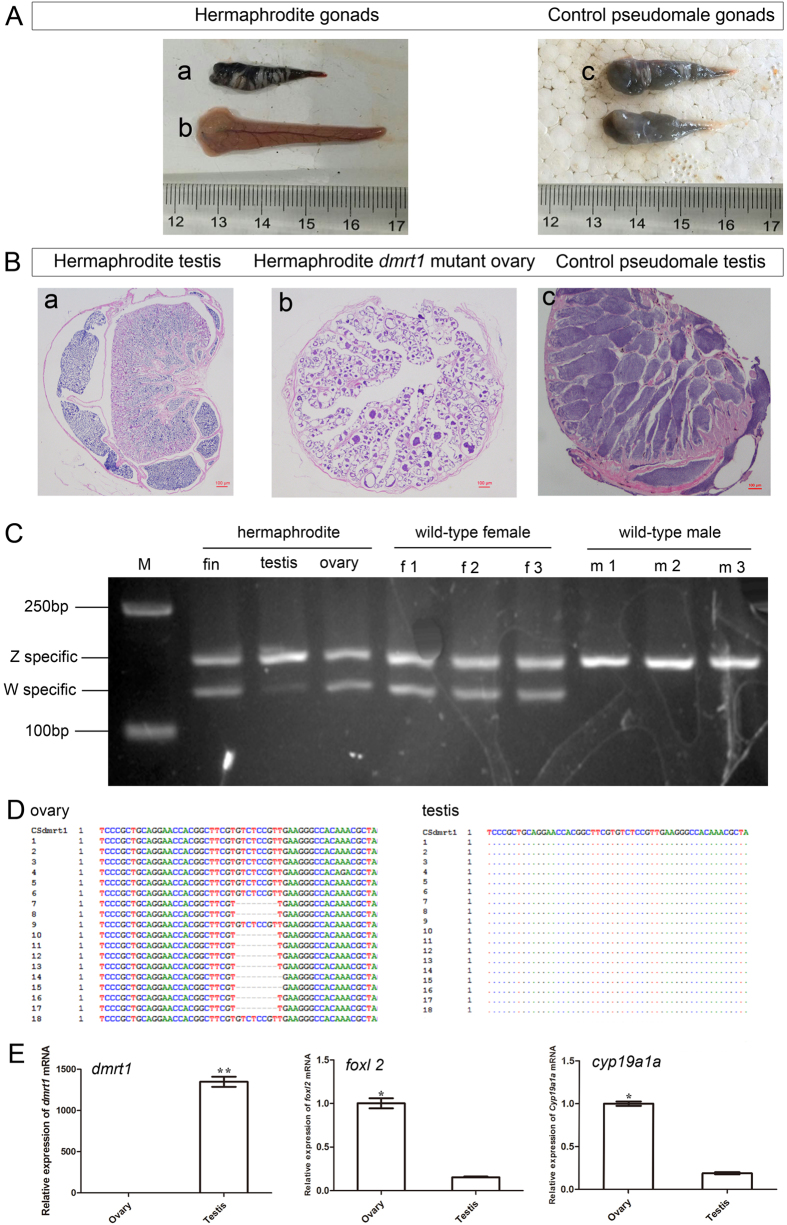
Effect of *dmrt1* disruption on intersex gonad phenotype, sex differentiation and gene expression. (**A**) Phenotype of gonads in the intersex. (a) Testis shaped up-side gonad of the intersex; (b) ovary shaped down-side mutant gonad of the intersex; (c) testes of a pseudomale. (**B**) Histology of the gonads. (a) Testis of the intersex showing normal male structures; (b) *dmrt1* mutant gonad (ovary) of the intersex; (c) testis of a pseudomale. Scale bar, 100 μm. (**C**) Determination of the genetic sex of the intersex by SSR PCR yielding different sized products for the Z (169 bp) and W (134 bp) chromosomes. (**D**) Sequences of wild-type and mutated target sites of *dmrt1* retrieved from ovary, partial; and only wild-type *dmrt1* in testis, partial. (**E**) Relative mRNA expression of *dmrt1, foxl2* and *cyp19a1a* in the *dmrt1*-deficient gonad at one year of age. *β-actin* was used as internal standard.

Among the 33 *dmrt1* mutated tongue soles we discovered a intersex with a testis on the up-side and an ovary on the down-side of the body (Fig. 6A). Histological examination demonstrated that the upside gonad has a typical testis structure with abundant spermatozoa, while the down-side gonad has a typical ovary structure and contains many oocytes (Fig. 6B). The genetic sex of the intersex was identified to be ZW by sex linked SSR marker analysis. Usually ZW genotypes develop as phenotypic females but some can develop as phenotypic males (pseudomales or neo-males). Notably, PCR products from fin and ovary of the intersex have the same abundance of the W and the Z derived fragment like in control females (Fig. 6C), while amplification from the testis gave a lighter W-specific band (Fig. 6C). As Z and W are of the same dosage in this fish the two sex linked SSR bands should be equal in DNA content. The weaker band for the W-linked marker might be due to the fact that sperm cells in the testis contain only Z-chromosomes because spermatocytes with a W-chromosome do not develop^11^.

Sequencing of cloned *dmrt1* PCR products revealed *dmrt1* mutations in the ovary with a mutation rate of 56%. No *dmrt1* mutation was detected in the testis of the intersex (Fig. 6D and Figure S6). This shows that the hermaphroditic fish is a chimera for the TALEN induced *dmrt1* mutation with the wildtype part developing as a pseudomale with a normal testis while the mutant part develops towards female with an ovary as a consequence of *dmrt1* disruption. By qPCR, *dmrt1* expression was only detected in the testis, but not in the ovary (Fig. 6E and Figure S7A). And *foxl2* and *cyp19a1a* gene expression in ovary was significantly higher than in testis (Fig. 6E and Figure S7B,C), which is consistent with the expression pattern in wild-type testis and ovary (Fig. 4).

## Discussion

Genome editing techniques have recently been widely applied in model fish such as zebrafish and medaka, and also several other freshwater fish, such as tilapia^18^,^19^,^21^. In marine fish transgenic technologies have lagged behind because of the almost insurmountable problems presented by the general fragility of the embryos and high mortalities. In the present study, we developed a microinjection technique for flatfish embryos and successfully produced a gene knock out by TALEN mediated genome editing in a marine fish species.

*Dmrt1* is a transcription factor that plays an important role in testis determination and differentiation in vertebrates^5^,^11^,^25^,^26^,^27^. In the mouse, *dmrt1* null mutants have severely dysgenic testes in which both Sertoli cells and germ cells fail to differentiate properly after birth^28^,^29^. In chicken where *dmrt1* is located on the Z chromosome^30^, modulation of expression levels showed that this gene is necessary and sufficient for male development^5^,^31^. In several fish species, including medaka, where a duplicate of *dmrt1* is the master male sex determining gene^32^, over-expression of *dmrt1* resulted in masculinization, while knock-down or knock-out induced feminization^33^,^34^. Here we show that *dmrt1* deficiency due to a TALEN generated gene knockout in *C. semilaevis* led to significantly compromised testis development and hypoplasia of the testes, and an overall macroscopic female-like structure of the gonad. We even detected oocyte structures in the mutant gonads indicating a high degree of feminization brought about by the lack of *dmrt1* expression. In addition, mutant *dmrt1* fish showed also feminized growth traits. As faster growth and higher body weight is a secondary sex character in tongue sole, this feature of the mutant fish is most likely a consequence of the sex reverted gonad and indicated that also the hormonal status of the mutant gonads was feminized.

In all previous experiments conducted so far in any vertebrate *dmrt1* deficiency was incompatible with normal differentiation and development of the testis. *Dmrt1* gene mutation of medaka, human and birds resulted in male-to-female sex reversal^5^,^35^,^36^. A few spermatogonia were still observed in *dmrt1*-deficient testis of *C. semilaevis*, but few or even no spermatocytes and spermatids. The observation that in most individuals no full gonadal sex reversal was seen may be due to the fact that these fish were of the G0 generation and that they constitute a mixture of cells with homozygous and heterozygous mutant loci, and that even some wildtype cells could still be present. This possibility is supported by the detection of a intersex mosaic fish in the cohort of tongue soles that developed from the injected embryos.

So far, a intersex has not been discovered in tongue sole in nature. The intersex obtained in this study was of ZW genotype with the *dmrt1* gene being mutated in the ovary, but no *dmrt1* mutation in the testis. Under hatchery conditions genetic female soles with ZW sex chromosomes can spontaneously develop into phenotypic males, which are called pseudomales or neo-males^11^. Thus, one explanation is that the intersex would be such a neo-male and would have developed with two testis, one on the up-side and one on the down-side. But due to the *dmrt1* mutation in the down-side part of the flatfish the gonad in this region developed into ovary.

Interestingly, we observed in the chimeric intersex the most complete sex reversal. This may be due to the genomic constitution of the pseudo-males, which are hemizygous for the Z-chromosome and thus have only a single copy of the *dmrt1* gene. In this situation the effect of the TALEN knockout of this gene should be more effective and thus apparent in the injected G0 individual.

In vertebrates, *dmrt1*, on the one side and *foxl2* and *cyp19a1a* on the other side are antagonistically expressed and cross-regulate each other during gonad development and in maintaining the identity of the adult ovary or testis^37^,^38^,^39^. Consistent with numerous studies in other vertebrates^21^,^40^,^41^, *foxl2* and *cyp19a1a* expression were significantly up-regulated and *Sox9a* and *Amh* expression were significantly down-regulated in *dmrt1-*deficient testes of *C. semilaevis* as a molecular signature of the feminization process in the mutant gonads.

In our previous studies real-time PCR analysis, methylation status across the differentially methylated region between males and females, and expression studies of *dmrt1* in normal testis and ovary of *C. semilaevis* revealed that *dmrt1* of *C. semilaevis* has convergent features that are compatible with a similar function determining male as in birds^11^. The successful knock-out of the Z-linked *dmrt1* gene in tongue sole reported here, which resulted in female development, confirmed our previous hypothesis^11^ that *dmrt1* is an important male-determining gene in *C. semilaevis.*

As a secondary effect, the mutant fish showed an enhanced growth as a typical female feature. As this is an important trait of high economic importance for the aquaculture industry, future studies will reveal the relationships between *dmrt1* as a primary male sex determining gene and its secondary functions on growth and body size.

In our study, we have developed methods for mRNA, DNA or anti-sense oligonucleotide microinjection into *C. semilaevis* embryos. The technique can be applied for studying many developmental processes *in vivo* and the functions of genes in flatfish. We successfully applied a genome editing technology to marine fish for the first time to study sex determination mechanism and to produce favorable economic traits, thus opening new avenues in the application of genome editing nucleases in both basic and applied research.

In conclusion, we have confirmed that *dmrt1* is an essential gene in determining male sexual development of *C. semilaevis*. To further show that it is the single master male sex-determining gene in tongue sole evidence could be provided that this gene is also sufficient to induce male development, e.g. by transgenic expression in WZ embryos. A situation in which *dmrt1* is the male sex determining gene in this fish analogous to the situation in birds will support the hypothesis (known as the “some chromosomes and some gene are better at sex” hypothesis) that certain genes and ancestral chromosomes are repeatedly evolutionary “selected” to serve as master regulators of genetic sex determination^42^.

## Materials and Methods

### Ethics statement

The collection and handling of the animals in the study was approved by the Chinese Academy of Fishery Sciences’ animal care and use committee, and all experimental animal protocols were carried out in accordance with the guidelines for the care and use of laboratory animals at the Chinese Academy of Fishery Sciences.

### Experimental fish production and sampling

*C. semilaevis* were kept in the Haiyang High-Tech Experimental Base (Haiyang, China). The parental fish were strengthening reared and spawned by induction in seawater at 21–23 °C. Zygotes were obtained by artificial insemination and cultivated in sterilized seawater at 22–23 °C.

**Figure 7:**
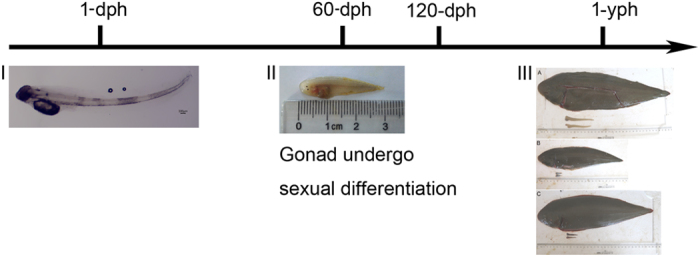
The Schedule of sampling. Sexual differentiation happens at approximately 60-dph. The tissues for gonadal histology and gene expression studies were sampled at about approximately 1-yph. (I) 1-dph C. semilaevis. (II) 60-dph C. semilaevis. (III) 1-yph C. semilaevis. (A) Wild-type female C. semilaevis; (B) wild-type male C. semilaevis; (C) dmrt1-deficient male C. semilaevis.

In Chinese tongue sole, sexual differentiation happens at ~60 days after hatching (60-dph). The tissues for gonadal histology and gene expression studies were sampled at about 1 year after hatching. Owing to the limited number of the injected fish obtained and the 60-dph gonads being sampled difficultly, we have to skip the 60-dph sample and only sampled at 1-yph (Fig. 7).

### Development of microinjection techniques for tongue sole embryos

Because so far no methods are available for embryo microinjection in flatfish, the corresponding techniques had to be developed for tongue sole.

The *smyd1*:*gfp* plasmid (50 ng/μl) driving GFP expression by a 5.3-kb muscle-specific *smyd1* promoter fragment^22^ was microinjected into tongue sole embryo at the 1–4 cell stage using a microinjector system (PV820, WPI, USA). For the injection procedure, tongue sole embryos were held in troughs (width: 0.95 mm ± 0.05 mm, depth: 1 mm ± 0.05 mm) made with a plexiglass mould in 1% agarose and aligned using forceps. The chorion of tongue sole embryos is very tough, thus thick pointed injection needles were prepared using a P-97 Flaming/Brown micropipette puller (Sutter Co, USA) (Fig. S1). The microinjection method is shown in Fig. 1A and suppl 1. In order to avoid the occurrence of reflux during microinjection, the holding pressure of the injector was adjusted to 0.1~3 psi (pounds per square inch). To avoid injury of the zygotes by excessive compression, special care was taken that they were not in the troughs for more than 3 minutes. The injected zygotes were transferred from the troughs to aseptic seawater (2 μl 1% methyleneblue in 100 ml aseptic seawater). ~1000 embryos in a 500 ml beaker with aseptic seawater were placed in a 22 °C–23 °C incubator. The injected embryos were transferred from the incubator to 30 L glass fiber-reinforce plastic tank and hatched in 22 °C–23 °C filtered seawater. The inflating volume was 0.9 L/min-3L/min. GFP fluorescence was observed and documented using a Nikon Eclipse 80i microscope (Nikon, Japan).

### Design and construction of *dmrt1*-TALENs

The target sites were selected following routine rules and the TALENs for *Csedmrt1* were constructed using the Golden Gate method as described previously^43^. Briefly, the modular plasmids recognizing each nucleotide were digested and ligated into the backbones of two middle array plasmids. Then the middle array plasmids and the last repeat plasmid were cloned into the backbones of the two optimized TALEN expression plasmids (the pCS2-TALEN-ELD and pCS2-TALEN-KKR) developed by the team of H. Cheng^44^.

The TALEN target sites of *Csedmrt1* are located in exon 1 (Fig. 2A) with the following recognition sequences: left TALEN 5′-TCCCGCTGCAGGAACCAC-3′ and right TALEN 5′-GAAGGGCCACAAACGCTA-3′, leaving between the two binding sites a 17 bp spacer sequence for cutting by the *Fok* I nuclease (Fig. 2A).

### TALEN mRNA preparation and microinjection

Plasmids were prepared using a plasmid midi kit (Omega, USA). pCS2-TALEN-ELD and pCS2-TALEN-KKR were linearized with *Not* I and recovered as transcription templates with a gel extraction kit (ZYMO RESEARCH, USA). *In-vitro* transcription was performed with the Sp6 mMESSAGE mMACHINE Kit (Ambion, USA). The mRNA was purified with the MEGAclear Kit (Ambion, USA) and RNA concentration was determined with NanoVue plus (Thermo Scientific, USA). The TALEN mRNAs were mixed yielding a final concentration of each arm of 100 ng/μl. TALEN mRNAs were stored at −80 °C. Microinjection of 100–300 pg of TALEN mRNAs into one to four cell stage embryos was conducted as described above.

### Determination of TALEN activity, mutation analysis, *dmrt1-*knockout fish screening and genetic sex identification

For an initial test of TALEN activity, injected embryos were collected at hatching stage. DNA was extracted with the TIANamp Marine Animals DNA Kit (TIANGEN, China) and quantified using NanoVue plus. A *dmrt1* fragment containing the TALEN target sites was amplified with primers *dmrt1*-TALEN-F and *dmrt1*-TALEN-R (Table S1). PCR, *T7E* I analyses, cloning and sequencing followed standard protocols (see Suppl 2).

Small pieces of tail fin from 6 months old injected *C. semilaevis* were used for mutant founder screening with the *T7E* I assay and sequencing as described in Suppl 2. According to the sequencing results, injected fish with high mutation rate were selected. Gonads were dissected from three *dmrt1-*deficient fish. The mutation rate of each gonad tissue was calculated as described in Suppl 2. Genetic sex of tongue sole was identified by PCR with the primers described previously^45^.

### Growth trait analyses of *dmrt1-*deficient *C. Semilaevis*

Body length and width of *dmrt1-*deficient *C. semilaevis* and control female and male fish were recorded. The weight was determined with an electronic scale.

### Histological and RT-PCR analyses of *dmrt1* knockout individuals

Histological sections of *dmrt1-*deficient fish (1 year old) and control fish were performed. Gonads were dissected and fixed in Bouin’s solution for 12 h~16 h at 4 °C, and stored in 70% ethanol. For sectioning, tissues were dehydrated and embedded in paraffin. Samples were serially sectioned at 6~8 um thickness and stained using hematoxylin-eosin (HE).

Total RNA was extracted from gonads of *dmrt1-*deficient and control fish using RNAfast200 Kit (FASTAGEN, China). DNase I treatment and cDNA preparation for RT-PCR and real-time PCR were carried out according to the suppliers instructions (Takara) using 7500 Real-Time PCR System (Applied Biosystems, USA). *Dmrt1, foxl2, cyp19a1a, Sox9a* and *Amh* mRNA expression were determined by RT-PCR and real-time PCR in three replicates. *β-actin* was used as the internal control^46^. Relative abundance of transcripts was calculated as R = 2^−ΔΔCt^. Primer sequences used for RT-PCR and real-time PCR are listed in Table S1. In addition, a forward primer was designed from the target sequence of *dmrt1* TALEN for identifying mutant fish by lower abundance of the expected product.

### Data analysis

Data were tested using one way ANOVA followed by Duncan multiple comparison tests with GraphPad Prism 5.0 (GraphPad, USA), significance was set at *p* < 0.05. Histograms were generated by GraphPad Prism 5.0 (GraphPad, USA).

## Additional Information

**How to cite this article:** Cui, Z. *et al*. Genome editing reveals *dmrt1* as an essential male sex-determining gene in Chinese tongue sole (*Cynoglossus semilaevis*). *Sci. Rep.* **7**, 42213; doi: 10.1038/srep42213 (2017).

**Publisher's note:** Springer Nature remains neutral with regard to jurisdictional claims in published maps and institutional affiliations.

## Supplementary Material

Supplementary Information

## References

[b1] Herpin A. & Schartl M. Plasticity of gene-regulatory networks controlling sex determination: of masters, slaves, usual suspects, newcomers, and usurpators. Embo Rep 16, 1260 (2015). 26358957 10.15252/embr.201540667 PMC4766460

[b2] Sinclair A. H. . A gene from the human sex-determining region encodes a protein with homology to a conserved DNA-binding motif. Nature 346, 240 (1990). 1695712 10.1038/346240a0

[b3] Gubbay J. . A gene mapping to the sex-determining region of the mouse Y chromosome is a member of a novel family of embryonically expressed genes. Nature 346, 245–250 (1990). 2374589 10.1038/346245a0

[b4] Shetty S. H., Kirby P. J., Zarkower D. & Graves J. A. M. *DMRT1* in a ratite bird: evidence for a role in sex determination and discovery of a putative regulatory element. Cytogenet Genome Res 99, 245–251 (2003). 10.1159/000071600 12900571

[b5] Smith C. A. . The avian Z-linked gene *DMRT1* is required for male sex determination in the chicken. Nature 461, 267 (2009). 19710650 10.1038/nature08298

[b6] Matsuda M. . *DMY* is a Y-specific DM-domain gene required for male development in the medaka fish. Nature 417, 559 (2002). 12037570 10.1038/nature751

[b7] Kamiya T. . A trans-species missense SNP in *Amhr2* is associated with sex determination in the tiger pufferfish, *Takifugu rubripes (fugu*). PLOS Genetics 8 (2012). 10.1371/journal.pgen.1002798 PMC3395601 22807687

[b8] Li M. . A Tandem Duplicate of Anti-Müllerian Hormone with a Missense SNP on the Y Chromosome Is Essential for Male Sex Determination in Nile Tilapia, *Oreochromis niloticus*. PLOS Genetics 11, e1005678 (2015). 26588702 10.1371/journal.pgen.1005678 PMC4654491

[b9] Zhou L. . The karyotype of the tonguefish *Cynoglossus semilaevis*. J. Fish Sci. China 3, 417–419 (2005).

[b10] Chen S. . Artificial gynogenesis and sex determination in half-smooth tongue sole (*Cynoglossus semilaevis*). Mar Biotechnol 11, 243–251 (2009). 18779997 10.1007/s10126-008-9139-0

[b11] Chen S. . Whole-genome sequence of a flatfish provides insights into ZW sex chromosome evolution and adaptation to a benthic lifestyle. Nat Genet 46, 253 (2014). 24487278 10.1038/ng.2890

[b12] Chen S. . Isolation of Female-Specific AFLP Markers and Molecular Identification of Genetic Sex in Half-Smooth Tongue Sole (*Cynoglossus semilaevis*). Mar Biotechnol 9, 273–280 (2007). 17308998 10.1007/s10126-006-6081-x

[b13] Chen S. . Induction of mitogynogenetic diploids and identification of WW super-female using sex-specific SSR markers in half-smooth tongue sole (*Cynoglossus semilaevis*). Mar Biotechnol 14, 120–128 (2011). 21735350 10.1007/s10126-011-9395-2

[b14] Ji X., Liu H., Chen S., Jiang Y. & Tian Y. Growth differences and dimorphic expression of growth hormone (GH) in female and male *Cynoglossus semilaevis* after male sexual maturation. Marine Genomics 4, 9–16 (2011). 21429460 10.1016/j.margen.2010.11.002

[b15] Song W. . Construction of a high-density microsatellite genetic linkage map and mapping of sexual and growth-related traits in half-smooth tongue sole (*Cynoglossus semilaevis*). Plos One 7 (2012). 10.1371/journal.pone.0052097 PMC3527371 23284884

[b16] Shao C. . Epigenetic modification and inheritance in sexual reversal of fish. Genome Res 24, 604–615 (2014). 24487721 10.1101/gr.162172.113 PMC3975060

[b17] Holleley C. E. . Sex reversal triggers the rapid transition from genetic to temperature-dependent sex. Nature 523, 79 (2015). 26135451 10.1038/nature14574

[b18] Hwang W. Y. . Efficient genome editing in zebrafish using a CRISPR-Cas system. Nat Biotechnol 31, 227 (2013). 23360964 10.1038/nbt.2501 PMC3686313

[b19] Ansai S. & Kinoshita M. Targeted mutagenesis using CRISPR/Cas system in medaka. Biology open, O20148177 (2014). 10.1242/bio.20148177 PMC4021358 24728957

[b20] Yano A. . An Immune-Related Gene Evolved into the Master Sex-Determining Gene in Rainbow Trout, *Oncorhynchus mykiss*. Curr Biol 22, 1423–1428 (2012). 22727696 10.1016/j.cub.2012.05.045

[b21] Li M. . Antagonistic Roles of *Dmrt1* and *Foxl2* in Sex Differentiation via Estrogen Production in Tilapia as Demonstrated by TALENs. Endocrinology (2013). 10.1210/en.2013-1451 24105480

[b22] Yamamoto Y. . Establishment of a stable transgenic strain in a pelagic egg spawning marine teleost, Nibe croaker *Nibea mitsukurii*. Aquaculture 313, 42–49 (2011).

[b23] Dong X., Chen S., Ji X. & Shao C. Molecular cloning, characterization and expression analysis of *Sox9a* and *Foxl2* genes in half-smooth tongue sole (*Cynoglossus semilaevis*). Acta Oceanologica Sinica 30, 68–77 (2011).

[b24] Deng S., Chen S., Xu J. & Liu B. Molecular cloning, characterization and expression analysis of gonadal P450 aromatase in the half-smooth tongue-sole, *Cynoglossus semilaevis*. Aquaculture 287, 211–218 (2009).

[b25] Raymond C. S. . Evidence for evolutionary conservation of sex-determining genes. Nature 391, 691–695 (1998). 9490411 10.1038/35618

[b26] Shibata K., Takase M. & Nakamura M. The *Dmrt1* expression in sex-reversed gonads of amphibians. Gen Comp Endocr 127, 232–241 (2002). 12225764 10.1016/s0016-6480(02)00039-4

[b27] Kobayashi T., Kajiura-Kobayashi H., Guan G. & Nagahama Y. Sexual dimorphic expression of *DMRT1* and *Sox9a* during gonadal differentiation and hormone-induced sex reversal in the teleost fish Nile tilapia (*Oreochromis niloticus*). Dev Dynam 237, 297–306 (2008). 10.1002/dvdy.21409 18095345

[b28] Kim S., Bardwell V. J. & Zarkower D. Cell type-autonomous and non-autonomous requirements for *Dmrt1* in postnatal testis differentiation. Dev Biol 307, 314–327 (2007). 17540358 10.1016/j.ydbio.2007.04.046 PMC1995593

[b29] Krentz A. D. . The DM domain protein *DMRT1* is a dose-sensitive regulator of fetal germ cell proliferation and pluripotency. P Natl Acad Sci USA 106, 22323–22328 (2009). 10.1073/pnas.0905431106 PMC2799724 20007774

[b30] Nanda I. . 300 million years of conserved synteny between chicken Z and human chromosome 9. Nat Genet 21 (1999). 10.1038/6769 10080173

[b31] Lambeth L. S. . Over-expression of *DMRT1* induces the male pathway in embryonic chicken gonads. Dev Biol 389, 160 (2014). 24576538 10.1016/j.ydbio.2014.02.012 PMC4201592

[b32] Nanda I. . A duplicated copy of *DMRT1* in the sex-determining region of the Y chromosome of the medaka, *Oryzias latipes*. P Natl Acad Sci USA 99, 11778–11783 (2002). 10.1073/pnas.182314699 PMC129345 12193652

[b33] Wu G. . Testicular *dmrt1* is involved in the sexual fate of the ovotestis in the protandrous black porgy. Biol Reprod 86, 41 (2012). 22034528 10.1095/biolreprod.111.095695

[b34] Wang D. . *Doublesex-* and *Mab-3-*Related Transcription Factor-1 Repression of Aromatase Transcription, a Possible Mechanism Favoring the Male Pathway in Tilapia. Endocrinology 151, 1331 (2013). 10.1210/en.2009-0999 20056824

[b35] Masuyama H. . *Dmrt1* mutation causes a male-to-female sex reversal after the sex determination by *Dmy* in the medaka. Chromosome Res 20, 163 (2012). 22187367 10.1007/s10577-011-9264-x

[b36] Raymond C. S. . A Region of Human Chromosome 9p Required for Testis Development Contains Two Genes Related to Known Sexual Regulators. Hum Mol Genet 8, 989–996 (1999). 10332030 10.1093/hmg/8.6.989

[b37] Raymond C. S., Kettlewell J. R., Hirsch B., Bardwell V. J. & Zarkower D. Expression of *Dmrt1* in the genital ridge of mouse and chicken embryos suggests a role in vertebrate sexual development. Dev Biol 215, 208 (1999). 10545231 10.1006/dbio.1999.9461

[b38] Loffler K. A., Zarkower D. & Koopman P. Etiology of Ovarian Failure in Blepharophimosis Ptosis Epicanthus Inversus Syndrome: *FOXL2* Is a Conserved, Early-Acting Gene in Vertebrate Ovarian Development. Endocrinology 144, 3237 (2011). 10.1210/en.2002-0095 12810580

[b39] Guiguen Y., Fostier A., Piferrer F. & Chang C. Ovarian aromatase and estrogens: A pivotal role for gonadal sex differentiation and sex change in fish. Gen Comp Endocr 165, 352–366 (2010). 19289125 10.1016/j.ygcen.2009.03.002

[b40] Alam M. A., Kobayashi Y., Horiguchi R., Hirai T. & Nakamura M. Molecular cloning and quantitative expression of sexually dimorphic markers *Dmrt1* and *Foxl2* during female-to-male sex change in *Epinephelus merra*. Gen Comp Endocr 157, 75–85 (2008). 18452918 10.1016/j.ygcen.2008.03.018

[b41] Elzaiat M. . High-Throughput Sequencing Analyses of XX Genital Ridges Lacking *FOXL2* Reveal *DMRT1* Up-Regulation Before *SOX9* Expression During the Sex-Reversal Process in Goats. Biol Reprod 91, 153, 1–14 (2014). 10.1095/biolreprod.114.122796 25395674

[b42] Graves J. A. M. & Peichel C. L. Are homologies in vertebrate sex determination due to shared ancestry or to limited options. Genome Biology 11, 205 (2010). 20441602 10.1186/gb-2010-11-4-205 PMC2884537

[b43] Liu Y. . A highly effective TALEN-mediated approach for targeted gene disruption in *Xenopus tropicalis* and zebrafish. Methods 69, 58–66 (2014). 24556556 10.1016/j.ymeth.2014.02.011

[b44] Lei Y. . Efficient targeted gene disruption in *Xenopus* embryos using engineered transcription activator-like effector nucleases (TALENs). P Natl Acad Sci USA 109, 17484 (2012). 10.1073/pnas.1215421109 PMC3491516 23045671

[b45] Liu Y. . SCAR-transformation of sex-specific SSR marker and its application in half-smooth tongue sole (*Cynoglossus semilaevis*). Journal of Agricultural Biotechnology. 22, 6 (2014).

[b46] Li Z. . *β-Actin* is a useful internal control for tissue-specific gene expression studies using quantitative real-time PCR in the half-smooth tongue sole *Cynoglossus semilaevis* challenged with LPS or *Vibrio anguillarum*. Fish Shellfish Immun 29, 89 (2010). 10.1016/j.fsi.2010.02.021 20227507

