# Supplementary material for: Genome editing reveals dmrt1 as an essential male sex-determining gene in Chinese tongue sole (Cynoglossus semilaevis)
Source: Sci Rep. 2017 Feb 16;7:42213. doi: 10.1038/srep42213 (PMC5311979; doi:10.1038/srep42213)
Supplement: Supplementary Information [file srep42213-s1.pdf]

Supplementary Information for

Genome editing reveals *dmrt1* as an essential male sex-determining gene in Chinese tongue sole (*Cynoglossus semilaevis*)

Zhongkai Cui[1,2,3^], Yun Liu[4^], Wenwen Wang[1,2], Qian Wang[1,2], Ning Zhang[1,2], Fan Lin[1,2], Na Wang[1,2], Changwei Shao[1,2], Zhongdian Dong[1,2], Yangzhen Li[1,2], Yingming Yang[1,2], Mengzhu Hu[1,2], Hailong Li[1,2], Fengtao Gao[1,2], Zhanfei Wei[1,2], Liang Meng[1,2], Yang Liu[1,2], Min Wei[1,2], Ying Zhu[1,2], Hua Guo[1,2], Christopher H.K. Cheng[4*], Manfred Schartl[5*] & Songlin Chen[1,2*]

1 Key Lab of Sustainable Development of Marine Fisheries, Ministry of Agriculture; Yellow Sea Fisheries Research Institute, Chinese Academy of Fishery Sciences, Qingdao 266071, China,

2 Laboratory for Marine Fisheries Science and Food Production Processes, Qingdao National Laboratory for Marine Science and Technology, Qingdao 266273, China,

3 College of Fisheries and Life, Shanghai Ocean University, Shanghai 201306, China,

4 School of Biomedical Sciences, The Chinese University of Hong Kong, Shatin, N.T., Hong Kong, China; School of Biomedical Sciences Core Laboratory, The Chinese University of Hong Kong Shenzhen Research Institute, Shenzhen 518057, China.

5 Physiologische Chemie, University of Würzburg, Biozentrum, and Comprehensive Cancer Center Mainfranken, University Clinic Würzburg, D-97074 Würzburg, Germany and Texas A&M Institute for Advanced Studies and Department of Biology, Texas A&M University, College Station, Texas 77843, USA

^ The first two authors contributed equally to this paper.

* equally contributing senior authors and corresponding authors: S.C. (chensl@ysfri.ac.cn), M.S. (phch1@biozentrum.uni-wuerzburg.de) , and C.H.K.C (chkcheng@cuhk.edu.hk)

Suppl 1

Microinjection was done in three steps: First, because *C. semilaevis* embryos are floating, more than half of the medium was aspirated from the embryo holding device. Leaving too much fluid makes the embryos falling in the trough. Second, the embryos were rotated to a position where the cell can be injected by the microinjection needle perpendicularly. Third, the microinjection needle was placed close to a glass slide. The tip of the needle gently touched the rough surface of the glass slide, until a small amount of *dmrt1*-TALENs mRNA flew out because of pressure from the injector device.

Suppl 2

The PCR reaction contained 2μl gDNA (~50ng/μl) as template, 5μl 10x reaction buffer (with $MgCl^{2+}$), 4μl dNTPs, 0.5μl Takara Ex Taq DNA polymerase (Takara, Japan), 1.5μl of each primer (at 10μM) and 35.5μl ddH_2O to a total volume of 50μl. PCR conditions were one cycle at 95°C for 5 min, followed by 35 cycles at 95°C for 30 sec, 60°C for 30 sec, and 72°C for 30 sec, and a final elongation step at 72°C for 10 min. The PCR product was purified using Gel Extraction Kit (CWBIO, China). 5μl of purified PCR product added to 1.1μl 1x NEBuffer and 4.4μl ddH_2O were heated to 95°C for 5min and then cooled to room temperature. Then *T7E* I (NEB, USA) was added and incubated at 37°C for 30 min. The reaction

mixture was separated by agarose gel electrophoresis and detected by Gel Imaging System (Bio-Rad, USA). The purified PCR product was cloned into the PMD-18T vector (Takara, Japan) and transformed into Top10 competent cells. Individual bacterial colonies were randomly selected and screened by PCR in a total volume of 25μl as described above. Positive clones were sequenced and mutated sites compared to wild-type sequences. The mutation rate of each sample was calculated from the result of sequencing (mutated sequences/total sequences).

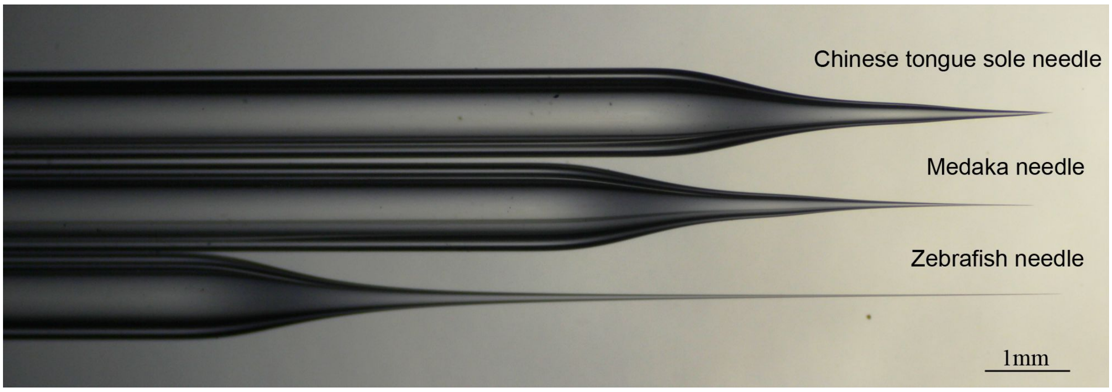

Figure S1 Comparison of *C. semilaevis,* medaka and zebrafish needles. The tip of a needle for embryo microinjection of *C. semilaevis* is shorter and thick pointed, to allow penetration of the tough chorion for injecting into the egg.

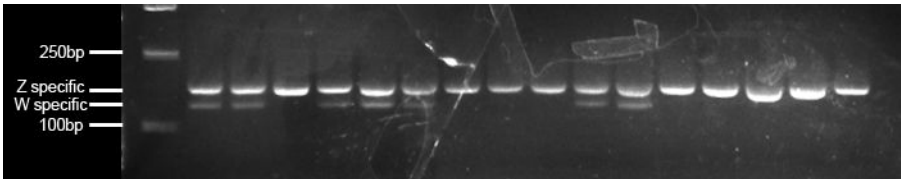

Figure S2 Determination of the genetic sex of the *dmrt1* mutated fish (partial). Different sized products for the Z (169 bp) and W (134 bp) chromosomes obtained by SSR PCR.

1 # ♀ 1R2R

CCCAAGTGCTCCCGCTGCAGGAACCACGGCTTCGTGTCTCCGTTGAAGGGCCACAAACGCTACT WT

CCCAAGTGCTCCCGTTGCAGGAA------------------------------------GGGCCACAAACGCTACT -24 1/5

2 # ♀ 2R3R

CCCAAGTGCTCCCGCTGCAGGAACCACGGCTTCGT----------GTCTCCGTTGAAGGGCCACAAACGCTACT WT

CCCAAGTGCTCCCGCTGCAGGAACCACGGCTTC-----------------------GTTGAAGGGCCACAAACGCTACT -8 2/5

CCC-----TCCAGTCACAGTAGC----GTTT-----------------------------GTTGAAGGGCCACAAACGCTACT (-38, +19) 1/5

CCCAAGTGCTCCCGCTGCAGGAACCACGGCTTCCGTTGAAGGAAGGCGTTGAAGGGCCACAAACGCTACT (-1, +5) 1/5

CCCAAGTGCTCCCGCTGCAGGAACCACGGCTTCGTTGAA----GGCTCCGTTGAAGGGCCACAAACGCTACT (-1, +5) 1/5

4 # ♂ 2R5R

CCCAAGTGCTCCCGCTGCAGGAACCACGGCTTCGTGTCTCCGTTGAAGGGCCACAAACGCTACT WT

CCCAAGTGCTCCCGCTGCAGGAACCACGGCT------------CCGTTGAAGGGCCACAAACGCTACT -8 2/5

5 # ♀ 3R4R

CCCAAGTGCTCCCGCTGCAGGAACCACGGCTTCGTGTCTCCGTTGAAGGGCCACAAACGCTACT WT

CCCAAGTGCTCCCGCTGCAGGAACCACGGCTTC------CTCCGTTGAAGGGCCACAAACGCTACT -4 1/5

7 # ♀ 4R5R

CCCAAGTGCTCCCGCTGCAGGAACCACGGCTTCGTGTCTCCGTTGAAGGGCCACAAACGCTACT WT

CCCAAGTGCTCCCGCTGCAGGAACCACGGCTTCG------------TTGAAGGGCCACAAACGCTACT -8 3/5

--TTGAAGGGCCACAAACGCTACT -42 1/5

9 # ♀ 2O3O

CCCAAGTGCTCCCGCTGCAGGAACCACGGCTTCGTGTCTCCGTTGAAGGGCCACAAACGCTACT WT

CCCAAGTGCTCCCGCTGCAGGAACCACGGCTTCCT------CCGTTGAAGGGCCACAAACGCTACT (-6, +2) 1/5

CCCAAGTGCTCCCGCTGCAGGAACCACGGCTCC-------------GTTGAAGGGCCACAAACGCTACT -8 1/5

CCCAAGTGCTCCCGCTGCAGGAACCACGGCTTC-------------GTTGAAGGGCCACAAACGCTACT (-10, +2) 2/5

10 # ♂ 2O4O

CCCAAGTGCTCCCGCTGCAGGAACCACGGCTTCGTGTCTCCGTTGAAGGGCCACAAACGCTACT WT

CCCAAGTGCTCCCGCTGCAGGAACCACGGCTTCTCGTCTCCGTTGAAGGGCCACCAACGGTACT (-2, +2) 1/5

CCCAAGTGCTCCCGCTGCAGGAACCACGGCT------------TCGTTGAAGGGCCACAAACGCTACT (-9, +1) 1/5

CCCAAGTGCTCCCGCTGCAG-----------------------------CCGTGGAAGGGCCACAAACGCTACT (-24, +5) 1/5

12 # ♂ 3O4O

CCCAAGTGCTCCCGCTGCAGGAACCACGGCTTCGTG------TCTCCGTTGAAGGGCCACAAACGCTACT WT

CCCAAGTGCTCCCGCTGCAGGAACCACGGCTTCGTGGGTCTCCGTTGAAGGGCCACAAACGCTACT +3 1/5

CCCAAGTGCTCCCGCTGCAGGAACCACGGCTTC----------------CGTTGAAGGGCCACAAACGCTACT -7 2/5

16 # ♂ 2R5O

CCCAAGTGCTCCCGCTGCAGGAACCACGGCTTCGTGTCTCCGTTGAAGGGCCACAAACGCTACT WT

CCCAAGTGCTCCCGCTGCAGGAACCACGGCC----------CTCCGTTGAAGGGCCACAAACGCTACT (-7, +1) 1/5

18 # ♀ 3R5O

CCCAAGTGCTCCCGCTGCAGGAACCACGGCTTCGTGTCTCCGTTGAAGGGCCACAAACGCTACT WT

CCCAAGTGCTCCCGCTGCAGGAACCACGGCT------------CCGTTGAAGGGCCACAAACGCTACT -8 1/5

19 # ♀ 1Y2Y

CCCAAGTGCTCCCGCTGCAGGAACCACGGCTTCGTGTCTCCGTTGAAGGGCCACAAACGCTACT WT

CCCAAGTGCTCCCGCTGCAGGAACCACGGCC----------TCCGTTGAAGGGCCACAAACGCTACT -7 1/5

CCCAAGTGCTCCCGCTGCAGGAACCACGGC------------TCCGTTGAAGGGCCACAAACGCTACT -8 3/5

CCCAAGTGCTCCCGCTGCAGGAACCACGGC------------TTCGTTGAAGGGCCACAAACGCTACT (-9, +1) 1/5

20 # ♂ 1Y3Y

CCCAAGTGCTCCCGCTGCAGGAACCACGGCTTCGT------GTCTCCGTTGAAGGGCCACAAACGCTACT WT

CCCAAGTGCTCCCGCTGCAGCAACCACGGCTTCC----------------GTTGAAGGGCCACAAACGCTACT (-8, +1) 1/5

CCCAAGTGCTCCCGCTGCAGGAACCACGGC--------------------------TTGAAGGGCCACAAACGCTACT -12 1/5

CCCAAGTGCTCCCGCTGCAGGAACCACGGCTTCGTTTGGCCTCCGTTGAAGGGCCACAAACGCTACT +4 1/5

CCCAAGTGCTCCCGCTGCAGGAACCACGGCTTCGGGTTGAACCCGTTGAAGGGCCACAAACGCTACT +7 1/5

21 # ♂ 1Y4Y

CCCAAGTGCTCCCGCTGCAGGAACCACGGCTTCGTGTCTCCGTTGAAGGGCCACAAACGCTACT WT

CCCAAGTGCTCCCGCTGCAGGAACCACGGCTTCGT------------TGAAGGGCCACAAACGCTACT -8 1/5

CCCAAGTGCTCCCGCTGCAGGAACCACGGCT------------CCGTTGAAGGGCCACAAACGCTACT -8 1/5

22 # ♂ 1Y5Y

CCCAAGTGCTCCCGCTGCAGGAACCACGGCTTCGTGTCTCCGTTGAAGGGCCACAAACGCTACT WT

CCCAAGTGCTCCCGCTGCAGGAACCACGGCT------------CCGTTGAAGGGCCACAAACGCTACT -8 1/5

23 # ♂ 2Y3Y

CCCAAGTGCTCCCGCTGCAGGAACCACGGCTTCGTGT----CTCCGTTGAAGGGCCACAAACGCTACT WT

CCCAAGTGCTCCCGCTGCAGGAACCACGGCTTCGTGTGTCTCCGTTGAAGGGCCACAAACGCTACT +2 1/5

CCCAAGTGCTCCCGCTGCAGGAACCACGGCTTCGT----------------TGAAGGGCCACAAACGCTACT -8 1/5

24 # ♂ 2Y4Y

CCCAAGTGCTCCCGCTGCAGGAACCACGGCTTCGTGTCTCCGTTGAAGGGCCACAAACGCTACT WT

CCCAAGTGCTCCCGCTGCAGGAACCACGGCT------------TCGTTGAAGGGCCACAAACGCTACT (-9，+1) 2/5

CCCAAGTGCTCCCGCTGCAGGAACCACGGCT------------CCGTTGAAGGGCCACAAACGCTACT -8 2/5

Figure S3 Wild-type sequence and *dmrt1* mutations of 16 injected individuals (partial). Mutation frequency ranging from 20% to 100%.

A

```
CCCAAGTGCTCCCGCTGCAGGAACCACGGCTTCGTGTCTCCGTTGAAGGGCCACAAACGCTACT   WT
CCCAAGTGCTCCCGCTGCAGGAACCACGGCTTCG-----CTCCGTTGAAGGGCCACAAACGCTACT   -3   1/12
CCCAAGTGCTCCCGCTGCAGGAACCACGGCT------------CCGTTGAAGGGCCACAAACGCTACT   -8   7/12
-----------------------------------------------------------------TCCGTTGAAGGGCCACAAACGCTACT   -97   2/12
```

B

```
CCCAAGTGCTCCCGCTGCAGGAACCACGGCTTCGTGTCTCCGTTGAAGGGCCACAAACGCTACT   WT
CCCAAGTGCTCCCGCTGCAGGAACCACGGCT------------TCGTTGAAGGGCCACAAACGCTACT   (-9，+1)   2/5
CCCAAGTGCTCCCGCTGCAGGAACCACGGCT------------CCGTTGAAGGGCCACAAACGCTACT   -8   2/5
```

Figure S4 Wild-type sequence and *dmrt1* mutations in the ovary (A) and fins (B) of the same fish. The frequency of *dmrt1* mutations was about 80% in this individual.

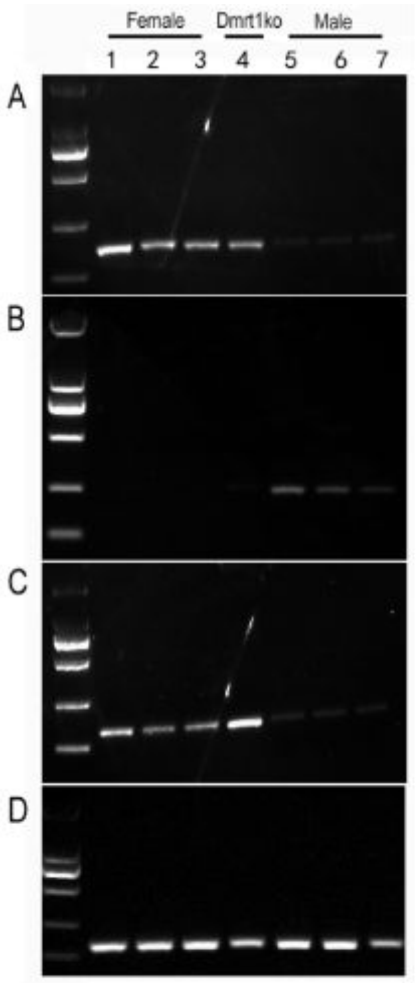

Figure S5 Expression of *dmrt1*, *foxl2* and *cyp19a1a* in *dmrt1*-deficient gonads of one year old fish determined by semiquantitavie RT-PCR. *β-actin* was used as control and for calibration of RNA amounts. **A**, mRNA expression of *cyp19a1a* in *dmrt1*-deficient testis is comparable to wt females, while only amplification barely above background is recorded in wild-type males. **B**, RT-PCR for *dmrt1* with RNA from *dmrt1*-deficient testis revealed considerably less amplification compared with wild-type males. **C**, mRNA expression of *foxl2* in *dmrt1*-deficient testis appears to be even higher in comparison with wild-type females. **D**, mRNA expression of *β-actin* in *dmrt1*-deficient tongue sole, wild-type females and wild-type males.

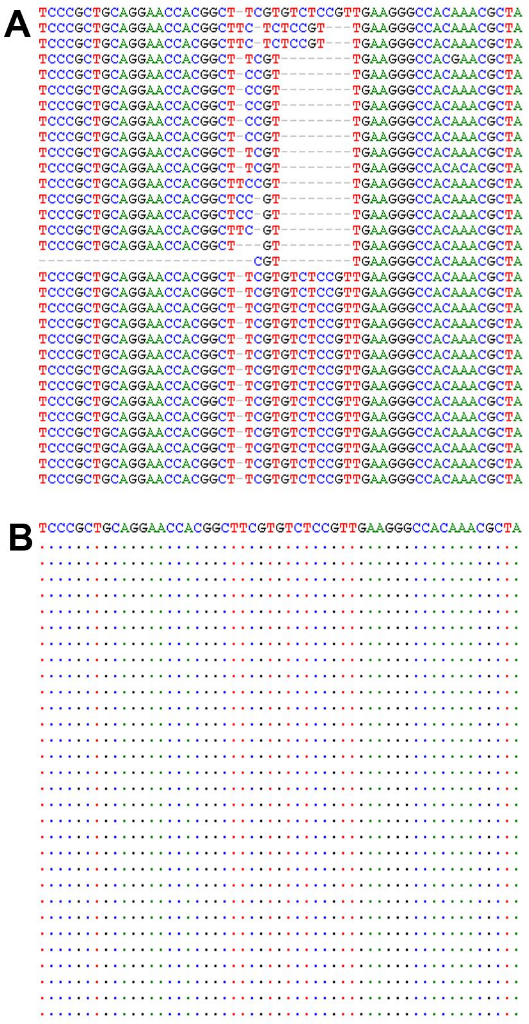

Figure S6 *Dmrt1* sequences of ovary and testis from the hermaphroditic fish. A, sequences of wild-type and mutated target sites of *dmrt1* retrieved from ovary, partial. **B**, sequences of only wild-type dmrt1 in testis, partial.

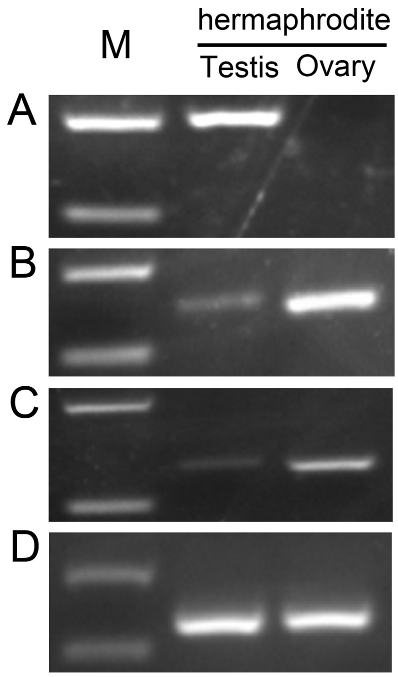

Figure S7 Expression of *dmrt1*, *fox12* and *cyp19a1a* in gonads of the hermaphroditic fish. *β-actin* was used as control and for calibration of RNA amounts. **A**, mRNA expression of *dmrt1* in intersexual testis is significantly higher than that in intersexual ovary. **B**, RT-PCR for *cyp19a1a* with RNA from intersexual testis revealed less amplification compared with intersexual ovary. **C**, mRNA expression of *foxl2* in intersexual ovary appears to be higher in comparison with intersexual testis. **D**, mRNA expression of *β-actin* in gonads of intersexual tongue sole.

Table S1. Primers used in this study.

Primer	Sequence (5'-3')	Purpose
dmrt1-TALEN-F	CGGGCAAAGGGAGAAGG	*dmrt1* amplification
dmrt1-TALEN-R	AAAAACATCTCCTGAGGGCTAA	
cs-sex-F	CCTAAATGATGGATGTAGATTCTGTC	determination of genetic sex
cs-sex-R	GATCCAGAGAAAATAAACCCAGG	
dmrt1-RT-F	CCGGACGGCTTCGTGTC	real-time PCR
dmrt1-RT-R	CTTCCACAGGGAGCAGGCAGT	
fox12-RT-F	GCACCCAATCCGTTCAGC	real-time PCR
fox12-RT-R	GCCATTTCGTCACCCTCT	
cyp19a1a-RT-F	CTCTGTTCCTCAGGTTTCTCTC	real-time PCR
cyp19a1a-RT-R	GATGTGACCCAGTGTGTGTTG	
Sox9a-RT-F	CAGGCAGGTAATGTTGGGGT	Real-time PCR
Sox9a-RT-R	AAGGAGCCGTAGGTGATGTG	
Amh-RT-F	ATGTGGCTGTAGAAGAAGAC	Real-time PCR
Amh-RT-R	CTGTTAGCAGGATGTATCG	

Table S2. Growth traits of *dmrt1* mutant and wild-type *C. semilaevis*.

type	2014 (1 year old)			2015 (8 months old)		
	Weight (g)	Body length (cm)	Body width (cm)	Weight (g)	Body length (cm)	Body width (cm)
WTF 1	335.7	40.3	11.3	134.1	27.4	8.4
WTF 2	259.7	32.9	9.0	104.5	25.5	7.5
WTF 3	295.1	34.5	9.7	105.2	25.8	7.5
DDM 1	250.5	34.0	9.5	120.0	26.7	7.7
DDM 2	202.4	32.6	9.0	116.2	26.5	7.7
DDM 3	270.7	34.4	10.5	102.1	26.0	7.5
WTM 1	113.2	25.4	7.4	53.2	21.0	6.0
WTM 2	100.6	25.6	6.7	61.8	22.3	6.4
WTM 3	117.0	27.3	7.0	52.6	21.0	6.0

WTF wild-type female *C. semilaevis*, DDM *dmrt1* mutant male *C. semilaevis*, WTM wild-type male *C. semilaevis*,
